# Preventive and therapeutic effect of *Lactobacillus paracasei* ZFM54 on *Helicobacter pylori*-induced gastritis by ameliorating inflammation and restoring gastric microbiota in mice model

**DOI:** 10.3389/fnut.2022.972569

**Published:** 2022-08-24

**Authors:** Qingqing Zhou, Nuzhat Qureshi, Bingyao Xue, Zuorui Xie, Ping Li, Qing Gu

**Affiliations:** Key Laboratory for Food Microbial Technology of Zhejiang Province, College of Food Science and Biotechnology, Zhejiang Gongshang University, Hangzhou, China

**Keywords:** Lactobacillus *paracasei* ZFM54, *Helicobacter pylori*, gastritis, gastric microbiota, inflammation cytokines

## Abstract

*Helicobacter pylori* is the most prevalent pathogen causing chronic gastritis, gastroduodenal ulcers, and gastric tumors and is asymptomatically present in 50% of the world's population. This research is focused on investigating the effect of *Lactobacillus paracasei* ZFM 54 (CCTCC NO:2016667) on attenuating *H. pylori*-induced gastritis. *H. pylori* ZJC03 isolated from a patient with gastritis harbored the virulence genes of *vacA* and *cagA* and was highly resistant to metronidazole (MIC > 256 μg/mL). *In vitro* analysis revealed that the potential anti-*H. pylori* characteristics of *L. paracasei* ZFM54 in terms of 65.57 ± 1.87% survival rate in simulated gastric juices at a pH of 2.0, 69.00 ± 2.73% auto-aggregation, 30.28 ± 2.24% co-aggregation, 70.27 ± 2.23% urease inhibition, and 57.89 ± 1.27% radical scavenging. In *H. pylori* infectious mice, *L. paracasei* ZFM54 pre- and post-treatment reduced the levels of malondialdehyde in liver tissues to 0.71 ± 0.04 nmol/mgprot (*p* < 0.05) and 0.70 ± 0.06 nmol/mgprot (*p* < 0.05), respectively. Glutathione levels were increased to 1.78 ± 0.02 μmol/gprot (*p* < 0.05) and 1.76 ± 0.52 μmol/gprot (*p* < 0.05), respectively. *L. paracasei* ZFM54 significantly inhibited *H. pylori*-mediated inflammation observed in gastric mucosal repair and downregulated the mRNA expression of pro-inflammatory cytokines IFN-γ, IL-1β, and IL-6 (*p* < 0.01). Importantly, *L. paracasei* ZFM54 increased Firmicutes and Actinobacteriota and decreased the relative abundance of bacterial taxa belonging to Campilobacterota and Proteobacteria. With the preventive and therapeutic administration of *L. paracasei* ZFM54, significant reductions in the average relative abundance of genera *Helicobacter, Muribaculum, Staphylococcus, Lachnospiraceae_NK4A136_group, Prevotellaceae_UCG-001, Alloprevotella*, and *Oscillibacter* were observed compared to infected mice. These findings suggest that *L. paracasei* ZFM 54 has the potential to protect against *H. pylori* infection by ameliorating inflammation and restoring the gastric microbiota.

## Introduction

*Helicobacter pylori* is the only bacterium reported to cause cancer and has been grouped as a class I carcinogen by the International Agency for Research on Cancer (IARC). *H. pylori* infection is the main risk factor for gastric duodenal ulcer, tumor, and lymphoma. Approximately, half of the population on the earth is infected with *H. pylori* (1). A high prevalence of *H. pylori* infection was reported in developing countries with approximately 80% prevalence in China and some states of South America and Eastern Europe (2). The major virulence genes of *H. pylori* are vacuolating cytotoxin A (*vac A*) and cytotoxin-associated gene A (*cagA*), which are closely related to pathogenesis (3). *VacA* exists in almost all *H. pylori*, including the signaling region (s1 and s2 alleles) and the middle region (m1 and m2 alleles), while *cagA*, regarded as a virulence marker, is positive in 60–70% of *H. pylori* strains (4). Treatment of *H. pylori* infection includes two antibiotics and a proton pump inhibitor (PPI), but the emergence of antibiotic resistance in *H. pylori* presents a great challenge (5). Side effects of antibiotics (nausea, vomiting, and diarrhea) often lead to patients' non-compliance. Recurrence of infection after treatment is also a threat, especially in high prevalence areas (6). The declining rate of successful treatment due to antibiotic resistance, side effects, and patients' non-compliance prompts researchers to seek alternative therapy to control the risk of *H. pylori* infection and associated gastroduodenal ulcers and tumors (7).

*Lactobacillus* species are the most commonly studied genera to have potential probiotic characteristics and have been extensively studied to control *H. pylori* infection (8, 9). In Deng et al.'s study, *H. pylori* could only infect specific pathogen-free mice, while pre-gavage with *L. paracasei* FJ861111.1 inhibited the colonization of *H. pylori* in gastric tissue (10). *L. paracasei* HP7 associated with extracts of *Perilla frutescens* and *Glycyrrhiza glabra* inhibited *H. pylori* with virulence genes such as *alpA, cagA, flaA*, and *ureA* and significantly reduced the infection rate of *H. pylori* in mice (11). *L. paracasei* strain 06TCa19 was shown to suppress *H. pylori*-mediated inflammatory chemokines of IL-8 and regulated the activation of normal T-cell expression and secretion in MKN45 cells (12).

The mechanism of action of *Lactobacillus* has been investigated, but the exact underlying mechanism is largely unknown. *Lactobacillus* has been demonstrated to inhibit the urease enzyme activity of *H. pylori* in *in vitro, in vivo*, and clinical trials (9). Inhibition of urease would not allow *H. pylori* to colonize the gastric mucosa and further protect from *H. pylori*-mediated inflammation. Secretion of antimicrobial substances (H_2_O_2_, lactic acid, bacteriocin, etc.), competition for adhesion receptors, co-aggregation ability, strengthening epithelial barrier, and immunomodulation are the main actions of *Lactobacillus* against pathogens (13, 14). *H. pylori* also induced oxidative damage to the host using its multiple antigens such as lipopolysaccharides and the type IV secretory system (15). As a consequence, neutrophils produce free radicals to combat *H. pylori* and cause the accumulation of different reactive oxygen species (ROS), which subsequently damage the host's cellular membranes producing malondialdehyde (MDA), which is a hallmark of an *H. pylori* infection (16). Furthermore, the production of anti-oxidant metabolites such as glutathione (GSH) and butyrate helps *Lactobacillus* block free radicals with glutathione peroxidase (GSH-Px) (17). Not all *Lactobacilli* possess probiotic potential, and even the differences occur within the same species; therefore, the characteristics of newly identified strains should be studied in detail.

The present study study is aimed at investigating the potential of *L. paracasei* ZFM54 (CCTCC NO:M 2016667) to ameliorate gastric inflammation, oxidative stress, and microbiota dysbiosis in the mice model by administering *L. paracasei* ZFM54 before (prevention) and after (therapy) *H. pylori* challenge. The probiotic potential of *L. paracasei* ZFM54 was first examined in *in vitro* experiments to get insight into the strain's efficacy to withstand the harsh gastric environment and inhibition against *H. pylori*.

## Materials and methods

### Bacterial culture

*L. paracasei* ZFM 54 isolated from healthy infant's feces was stored at the China Center for Type Culture Collection (Wuhan, China) and sub-cultured in DeMan–Rogosa–Sharpe (MRS) broth at 37°C for 24 h. Cell-free supernatant (CFS) was obtained by centrifuging at 8,000 ×*g* at 4°C for 15 min.

*H. pylori* ZJC03 (CCTCC NO: M 20211218) was provided by the Zhejiang University School of Medicine and was previously isolated from patients with gastritis. *H. pylori* was inoculated on Columbia Blood Agar (CBA) broth supplemented with sheep's blood (7%, v/v) (Hopebio, Qingdao, China) and antibiotics (10 mg/mL vancomycin, 5 mg/mL amphotericin, 2.5 units/mL polymyxin B, and 5 mg/mL trimethoprim) (Sangon Biotech, Shanghai, China). The plates were kept in an anaerobic box containing a micro-aerobic pack (Anaeropack-MicroAero, Mitsubishi, Japan) and incubated at 37°C for 48–72 h. For liquid culture, the bacteria were scraped off and sub-cultured in Brain Heart Infusion (BHI) broth in microaerophilic condition at 37°C for 48 h.

### Simulated gastric juice tolerance

The simulated gastric juice tolerance assay of *L. paracasei* ZFM54 was detected according to Charteris et al. (18) with some modifications. Bacterial cells were centrifuged (4°C, 8,000 ×*g*) for 10 min and suspended in an equal volume of PBS (pH 7) after 18–20 h of culture. The pH of 1 g/L pepsin (Sigma Aldrich, USA) was set to 2, 3, and 4, respectively. A mixture of 100 μL cell suspension, 300 μL NaCl solution (0.5% w/v), and 1 mL pepsin with different pH was cultured at 37°C. Aliquots were removed at 0 h, 2 h, and 4 h, diluted, and enumerated for viability by plate count. The survival rate (%) was calculated as below:


(1) $$\text{Survival rate }(\%)=\frac{\text{No}.\text{ of cells survived}}{\text{No}.\text{ of initial viable cells inoculated}}\times \text{100}.\;$$

### Auto-aggregation assay

Auto-aggregation of *L. paracasei* ZFM54 was assayed as reported by Ahire et al. (19) with slight modifications. The initial optical density of the cell suspension at 600 nm was set to 0.5 and incubated at 37°C for 4 h. The OD_600_ was recorded every 1 h. The auto-aggregation rate (%) was measured by the following formula:


(2) $$\text{Auto − aggregation }(\%)\text{=}\frac{\text{A0 − At}}{\text{A0}}\times \text{100},$$

where A0 is the absorbance at 0 h, and At is the absorbance at time t = 1, 2, 3, and 4 h.

### Virulence genes

Genomic DNA of *H. pylori* ZJC03 was extracted by genome extraction kits (Takara, Beijing, China) according to the manufacturer's instructions. Virulence genes of *cagA, vacA s1/s2*, and *vacA m1/m2* were amplified, and the primers were synthesized (Sangon, Shanghai, China) and are listed in Table 1. PCR cycling procedure is as follows: initial denaturation step at 95°C for 10 min; 35 cycles of denaturation at 95°C for 50 s, annealing at 55°C for 30 s, extension at 72°C for 30 s; final extension at 72°C for 5 min; and incubation at 4°C. The amplified products were verified by gel electrophoresis (1% agarose). The size of DNA fragment was estimated using a 500-bp DNA Marker.

**Table 1:** Primer sequences for virulence genes.

**Primer**		**DNA sequence (5^′^-3^′^)**	**DNA size (bp)**
*cagA*	F	TTGACCAACAACCACAAACCGAAG	183
	A	CTTCCCTTAATTGCGAGATTCC	
*vacA s1/s2*	F	ATGGAAATACAACAAACACAC	259/286
	A	CTGCTTGAATGCGCCAAAC	
*vacA m1/m2*	F	CAATCTGTCCAATCAAGCGAG	567/642
	A	GCGTCAAAATAATTCCAAGG	

F, forward; R, reverse.

### Antibiotic sensitivity assay

Susceptibility of *H. pylori* ZJC03 to amoxicillin, metronidazole, tetracycline, and clarithromycin was evaluated by the epsilometric method (E-test) (20). The E-test strip containing antibiotics was attached to the surface of the agar medium containing *H. pylori* (10^8^ CFU/mL) and cultured in a microaerophilic tank (85 N_2_, 10 CO_2_, and 5% O_2_) at 37°C for 48 h−72 h. The minimal inhibitory concentration (MIC) was determined by the point of the antibacterial ring and the strip.

### Co-aggregation assay

Co-aggregation ability was assayed according to Kos et al. (21). The cells were washedhree times with PBS (pH 7.0), and the initial optical density was set to 0.50 at 600 nm. Same volumes of *L. paracasei* ZFM54 (10^9^ CFU/mL) and *H. pylori* ZJC03 (10^8^ CFU/mL) cell suspension were mixed, vortexed for 10 s, and incubated at 37°C for 4 h. OD_600_ was measured every 1 h. The co-aggregation rate (%) was measured by the following formula:


(3) $$\text{Co − aggregation }(\%)\text{=}\frac{(\text{Ax + Ay})\text{ − 2}\times \text{At}}{\text{Ax + Ay}}\times \text{100},$$

where Ax is the absorbance of *L. paracasei* ZFM54 at 0 h, Ay is the absorbance of *H. pylori* at 0 h, and At is the absorbance of the mixture at 1, 2, 3, and 4 h.

### Anti-*H. pylori* activity *in vitro*

As previously described by Hirano et al., antibacterial activity was determined by the Oxford cup assay with slight modifications (22). *H. pylori* ZJC03 cell cultured in a semi-liquid BHI medium containing 7% sheep's blood was poured onto agar plates in sterilized Oxford cups. After solidification, the Oxford cups were pulled out to form wells, and 100 μL of *L. paracasei* ZFM 54 cell (10^9^ CFU/mL) in phosphate-buffered solution (PBS) and CFS was added into different wells.

### Urease inhibition assay

Urease activity was used as the activity index of *H. pylori* ZJC03 by the phenol red method (23) with modification. In a 96-well microtiter plate, the bacterial cultures were added: (i) 40 μL *H. pylori* cells (10^8^ CFU/mL) and 10 μL *L. paracasei* 54 cells (10^9^ CFU/mL), (ii) 40 μL *H. pylori* cells (10^8^ CFU/mL) and 10 μL *L. paracasei* ZFM54 CFS, and (iii) 40 μL *H. pylori* cells (10^8^ CFU/mL) and 10 μL PBS. The 96-well microtiter plate was incubated at 37°C for 48 h under microaerophilic condition (85 N_2_, 10 CO_2_, and 5% O_2_). Urease indicator (0.9% NaC1, 20 mmo1/L Urea, 14 μg/mL phenol red, pH 6.8) was added to each well, and the absorbance was measured at 540 nm. The inhibition rate was calculated by the following formula:


(4) $$\begin{array}{l} \text{Urease inhibition }(\%)\\ =\frac{\text{ Urease activity of }H.\;pylori\;-\text{ Urease activity of sample}}{\text{Urease activity of }H.\;pylori}\\ \times \text{100}.\; \end{array}$$

### Free radical scavenging activity

The anti-oxidant activity was assessed by the 2,2-diphenyl-1-picrylhydrazyl (DPPH) free radical scavenging activity according to Saravanakumar et al. (24) with some modifications. About 1 mL *L. paracasei* ZFM54 cell culture (10^9^ CFU/mL) and 1 mL CFS were cultured in combination with 1 ml DPPH ethanol solution (0.2 mM) respectively. Then, 1 mL DPPH mixed with 1 mL saline was used as the control. All tubes were vortexed and placed in the dark at room temperature for 30 min. The absorbance was measured at 517 nm. The DPPH scavenging activity (%) was calculated by the following formula:


(5) $$\text{Scavenging activity }(\%)\text{=}\frac{\text{A1 − A2}}{\text{A1}}\times \text{100},$$

where A_1_ is the absorbance of the control and A_2_ is the absorbance of the sample.

### Animal model

**Figure 1:**
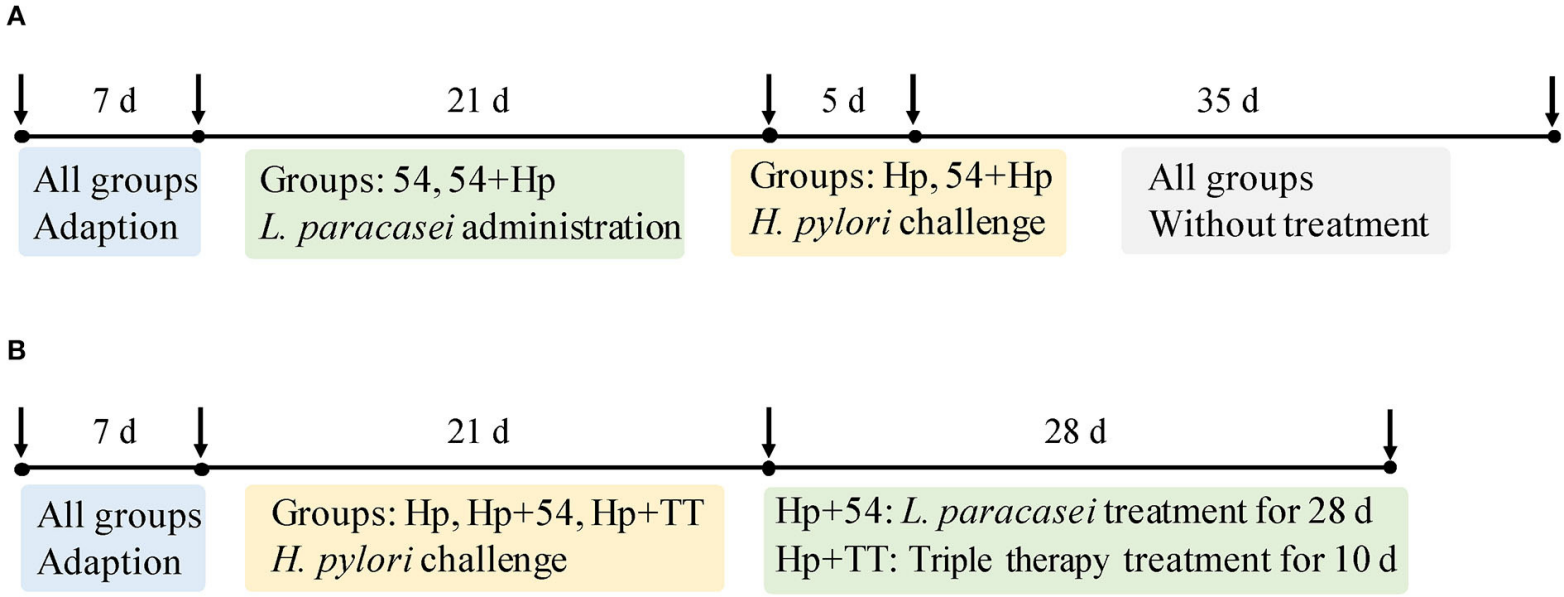
Grouping of C57BL/6 mice for prevention and therapeutic models. **(A)** The preventive groups of control (normal mice without prevention and infection in the preventive group), Hp (mice infected with *H. pylori* without prevention in the preventive group), 54 (only treated with *L. paracasei* ZFM54), and 54+Hp (pretreatment with *L. paracasei* ZFM54 before *H. pylori* challenge). **(B)** The therapeutic groups of control' (normal mice without prevention and infection in the therapeutic group), Hp' (mice infected with *H. pylori* without treatment in the therapeutic group), Hp+54 (treatment with *L. paracasei* ZFM54 after *H. pylori* challenge), and Hp+TT (treatment with triple therapy after *H. pylori* challenge).

The animal experiment was conducted in accordance with the ethical guidelines for animal use and with permission from Shanghai Public Health Clinical Center Animal Ethics Committee. Specific pathogen-free C57BL/6 mice (6-week-old, 18–20 g) were obtained from Sino-British SIPPR/BK Lab Animal Ltd. (Shanghai, China), and the experiment was carried out in the Laboratory Animal Department of Shanghai Public Health Clinical Center. Mice were adapted to the controlled condition (24 ± 2°C room temperature and 50 ± 5% relative humidity) for a week prior to the experiment. All mice were grouped into two experimental approaches including preventive administration and therapeutic administration, as seen in Figure 1. Six mice in each subgroup were randomly scarificed for sampling and testing.

#### Preventive trials

Mice in the control group (normal mice) were free to eat and drink without infection. On a group of 54 mice, with only *L. paracasei* ZFM54 (10^9^ CFU/mL) was administrated for 21 days without *H. pylori* challenge. After 21-day intragastrical administration of *L. paracasei* ZFM54, mice in the groups of Hp (negative control) and 54+Hp (prevention with *L. paracasei* ZFM54) were challenged with *H. pylori* ZJC03 (10^8^ CFU/mL, 400 μL) three times (every other day). After infection, all mice were fed with a normal diet for 35 days and finally sacrificed by cervical dislocation.

#### Therapeutic trials

Mice in the control' group (normal mice) were free to eat and drink without *H. pylori* ZJC03 infection. Mice in the groups of Hp' (negative control), Hp+54 (therapy with *L. paracasei* ZFM54), and Hp+TT (therapy with triple therapy) were challenged intragastrically with *H. pylori* (10^8^ CFU/mL, 400 μL) three times a week for 21 days. Then, mice in the groups of Hp+54 and Hp+TT were fed intragastrically with 400 μL *L. paracasei* ZFM54 (10^9^ CFU/mL) for 28 days and triple therapy for 10 days, respectively. Triple therapy (TT) consists of PPI (5.2 mg/kg), amoxicillin (260 mg/kg), and clarithromycin (65 mg/kg).

### MDA and GSH assays

Liver tissues of mice were homogenized and centrifuged at 4000 ×*g* for 10 min. The MDA level was determined using thiobarbituric acid (TBA) assay kit according to the manufacturer's instructions. One molecule of MDA reacts with two molecules of TBA to form thiobarbituric acid reactive substance (TBARS) at high temperature and acidic conditions and was measured at 532 nm. The following formula was used to calculate MDA content in liver tissues:


(6) $$\begin{array}{l} \text{MDA }\left(\text{nM}\right)\text{=}\frac{\text{measured OD value − controlled OD value}}{\text{standard OD value − blank OD value}}\\ \times \text{ Cs }\times \text{ Cp},\; \end{array}$$

where Cs is the standard concentration (10 nmol/mL), and Cp is the protein concentration of samples before being measured (mgprot/mL).

Tissue GSH level was determined using the GSH assay kit by the manufacturer's instructions. The reaction of 5, 5-Dithiobis(2-nitrobenzoic acid) (DTNB) with GSH takes place to produce a yellow chromogen, and the absorbance is measured at 405 nm. The calculation formula is as follows:


(7) $$\begin{array}{l} \text{GSH }\left(\text{mM}\right)\text{=}\frac{\text{measured OD value − blank OD value}}{\text{standard OD value − blank OD value}}\\ \times \text{ Cs }\times \text{ Cp }\times \text{ dilution factor of sample}, \end{array}$$

where Cs is the standard concentration (20 mM), Cp is the protein concentration of samples before being measured (mgprot/mL), and the dilution factor of samples is 2 times.

### Hematoxylin and eosin (H&E) staining

Histopathological alterations of gastric mucosa in preventive and therapeutic groups were evaluated by H&E staining. Gastric tissues of mice were fixed in 4% paraformaldehyde for 48 h at room temperature, were dehydrated, waxed, embedded, and sectioned according to Ahire et al. (19). H&E images were observed under a NIKON Eclipse Ci microscope assisted with a NIKON digital sight DS-FI2 (NIKON Corporation, Tokyo, Japan).

### Real-time quantitative polymerase chain reaction (RT-qPCR)

The mRNA expression of IL-1β, IFN-γ, IL-6, and IL-10 in the stomach tissues in the preventive and therapeutic groups was quantitatively analyzed by RT-qPCR (25). Total RNA from gastric tissue was extracted by TRIzol reagent (Servicebio, Wuhan, China) and reversed-transcribed into single-strand cDNA by RevertAid First Strand cDNA Synthesis Kit according to the manufacturer's instruction (Thermo Scientific, Vilnius, Lithuania). qPCR was conducted using FastStart Universal SYBR Green Master (Rox) (Servicebio, Wuhan, China) with the primers of inflammatory cytokines (Table 2). The cycling program consisted of initial denaturation at 95°C for 10 min followed by 40 cycles at 95°C for 15s, 60°C for 15 s, 60°C for 60 s, and 95°C for 15 s. The gene expression was calculated using the 2^−ΔΔCt^ method, with glyceraldehyde-3-phosphate dehydrogenase (GADPH) as the reference gene (21).

**Table 2:** Primers used for inflammatory cytokines.

**Gene**	**Primers**	**DNA sequences (5'-3')**	**Segment length (bp)**	**RefSeq ID**
GAPDH	S	CCTCGTCCCGTAGACAAAATG	133	NM_008084.2
	A	TGAGGTCAATGAAGGGGTCGT		
IFN-γ	S	CTCAAGTGGCATAGATGTGGAAG	251	NM_008337.4
	A	TGACCTCAAACTTGGCAATACTC		
IL-1β	S	TCAAATCTCGCAGCAGCACATC	206	NM_008361
	A	CGTCACACACCAGCAGGTTATC		
IL-6	S	CCCCAATTTCCAATGCTCTCC	141	NM_031168.2
	A	CGCACTAGGTTTGCCGAGTA		
IL-10	S	TTTAAGGGTTACTTGGGTTGCC	106	NM_010548.2
	A	AATGCTCCTTGATTTCTGGGC		

### 16S rRNA sequencing

The V3-V4 region of the 16S rRNA gene of gastric microbiota was sequenced using the QIIME pipeline (26). According to SILVA 138/16s database, paired-end reads were inferred by DADA2, linked to tags, and clustered to Amplicon Sequence Variant (ASV) with 100% similarity. Alpha diversity-related community richness (Chao index and Ace index) and community diversity (Shannon index and Simpson index) were analyzed. Beta diversity analysis was performed by principal coordinate analysis (PCoA) based on unweighted-uniFrac distances. The difference between the classes and the features such as organism, clades, and ASV was analyzed by the linear discriminant analysis effect size (LEfSe) method (LDA > 4.0). The relative abundance differences of the genus *Helicobacter* between the Hp group and the preventive or therapeutic groups were further analyzed.

### Statistical analysis

All results are expressed as mean value ± standard deviation (SD). Graphs of *in vitro* indexes, MDA, GSH, and inflammatory cytokines were drawn using GraphPad Prism 9.0, and the differences between the two groups were compared by Tukey's test or Student's t-test. The differences in gastric microbiota were analyzed using the Wilcoxon rank-sum test. A *p*-value < 0.05 was considered statistically significant.

## Results

### pH tolerance, auto-aggregation, and co-aggregation properties of *L. paracasei* ZFM54

**Figure 2:**
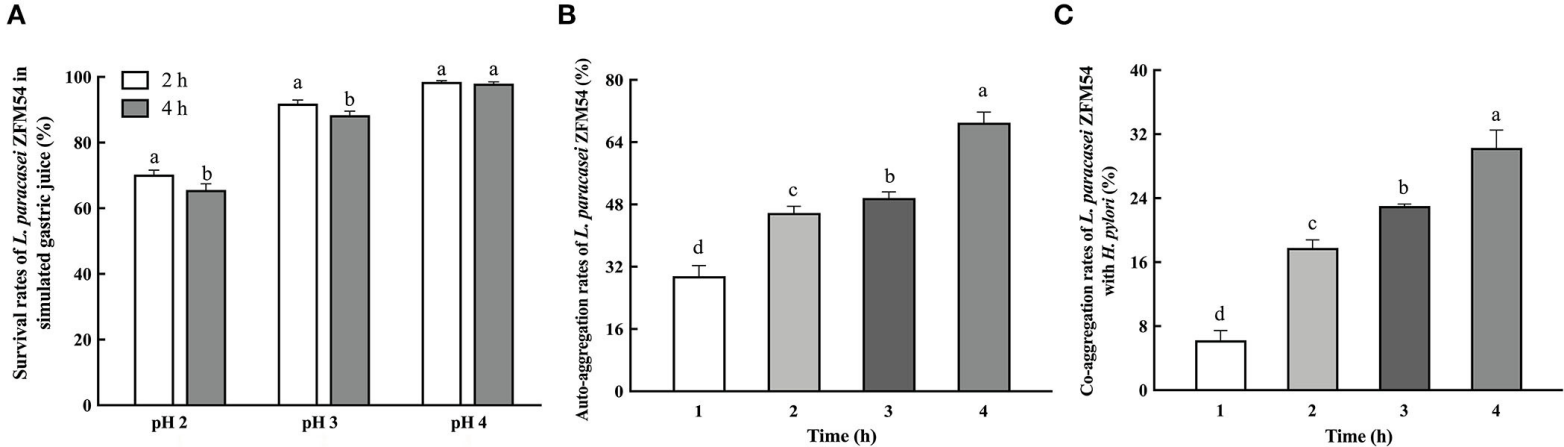
Tolerance to simulated gastric juice **(A)**, auto-aggregation rate **(B)**, and co-aggregation rate **(C)** of *L. paracasei* ZFM54. The results are expressed as mean ± standard deviation (*n* = 3), and columns with different lowercase letters were significantly different (*p* < 0.05) using Tukey's test.

Probiotic strains need to resist low pH to survive and colonize in the host's stomach. The survival rate of *L. paracasei* ZFM54 was 65.57 ± 1.87%, 88.37 ± 1.19%, and 97.99 ± 0.50% at pH 2, 3, and 4 for 4 h, respectively (Figure 2A). These findings indicated that *L. paracasei* ZFM54 can normally grow and meet the requirements for probiotic growth *in vivo*. The auto-aggregation ability of *L. paracasei* ZFM54 was checked, which is the ability to interact non-specifically with own cells. The auto-aggregation rate was 29.60 ± 2.73% after 1 h, which gradually increased to 45.80 ± 1.75% after 2 h and 69.00 ± 2.73% after 4 h (Figure 2B). *L. paracasei* ZFM54 was also able to co-aggregate with *H. pylori* ZJC03 *in vitro* at 6.23 ± 1.23% after 1 h, 17.78 ± 1.02% after 2 h, and 30.28 ± 2.24% after 4 h, respectively (Figure 2C).

### Virulence genes and antibiotic susceptibility of *H. pylori* ZJC03

**Figure 3:**
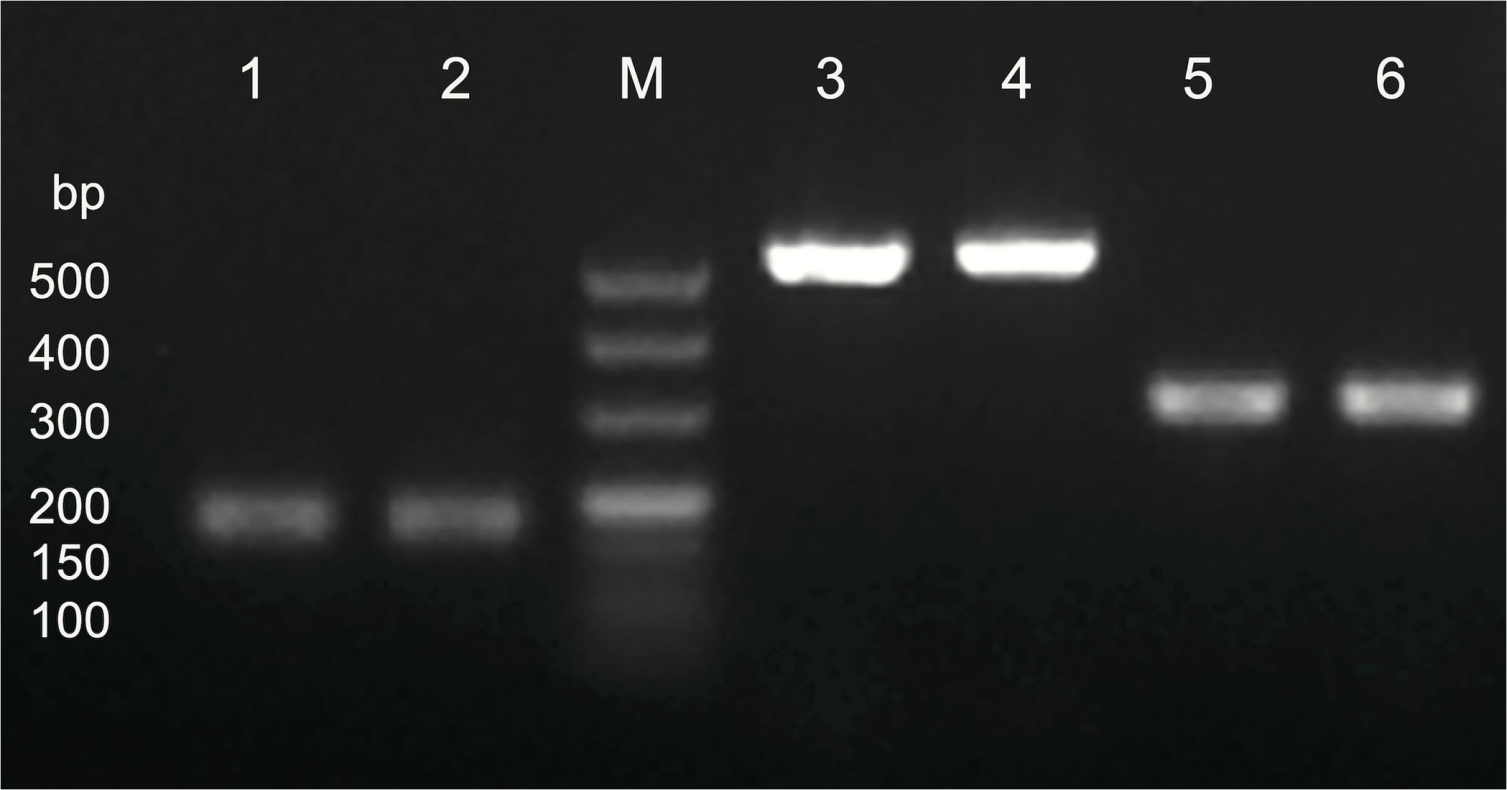
Virulence genes of *H. pylori* ZJC03. Lane M, maker; lane 1 and 2, *cagA*; lane 3 and 4, *vacA s1/s2*; lane 5 and 6, *vacA m1/m2*.

As seen in Figure 3, virulence genes of *cagA, vacA s1/s2*, and *vacA m1/m2* were all expressed in *H. pylori* ZJC03. E-test results showed that *H. pylori* ZJC03 was sensitive to clarithromycin, amoxicillin, and tetracycline with the MIC values of 0.80 μg/mL, 0.016 μg/mL, and 0.023 μg/mL, respectively. However, *H. pylori* ZJC03 was not sensitive to metronidazole, with an MIC value exceeding 256 μg/mL.

### Anti-*H. pylori* activity of *L. paracasei* ZFM54

The cells of *L. paracasei* 54 showed stronger anti-*H. pylori* ability compared to CFS. The inhibition zone of the cells was 11.25 ± 0.78 mm, and the zone of the CFS was 9.02 ± 0.20 mm (Table 3). *L. paracasei* ZFM 54 cells inhibited urease activity of *H. pylori* ZJC03 with a rate of 70.27 ± 2.23%, while CFS showed an increased inhibition rate of 89.51 ± 0.72%. *L. paracasei* ZFM54 exhibited strong radical scavenging ability in DPPH scavenging assay. *L. paracasei* ZFM 54 cells showed remarkable oxidative activity calculated to be 57.89 ± 1.27%, and CFS was 51.36 ± 0.78%.

**Table 3:** Anti-*H. pylori* activity, urease inhibition, and DPPH scavenging of *L. paracasei* 54.

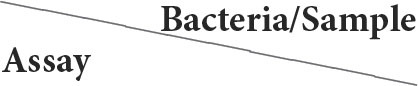	**Anti-*H. pylori* activity (mm)**	**Urease inhibition** **(%)**	**DPPH scavenging (%)**
Cells of *L. paracasei* 54	11.25 ± 0.78	70.27 ± 2.23	57.89 ± 1.27
CFS of *L. paracasei* 54	9.02 ± 0.20	89.51 ± 0.72	51.36 ± 0.78

### MDA and GSH levels in liver

**Figure 4:**
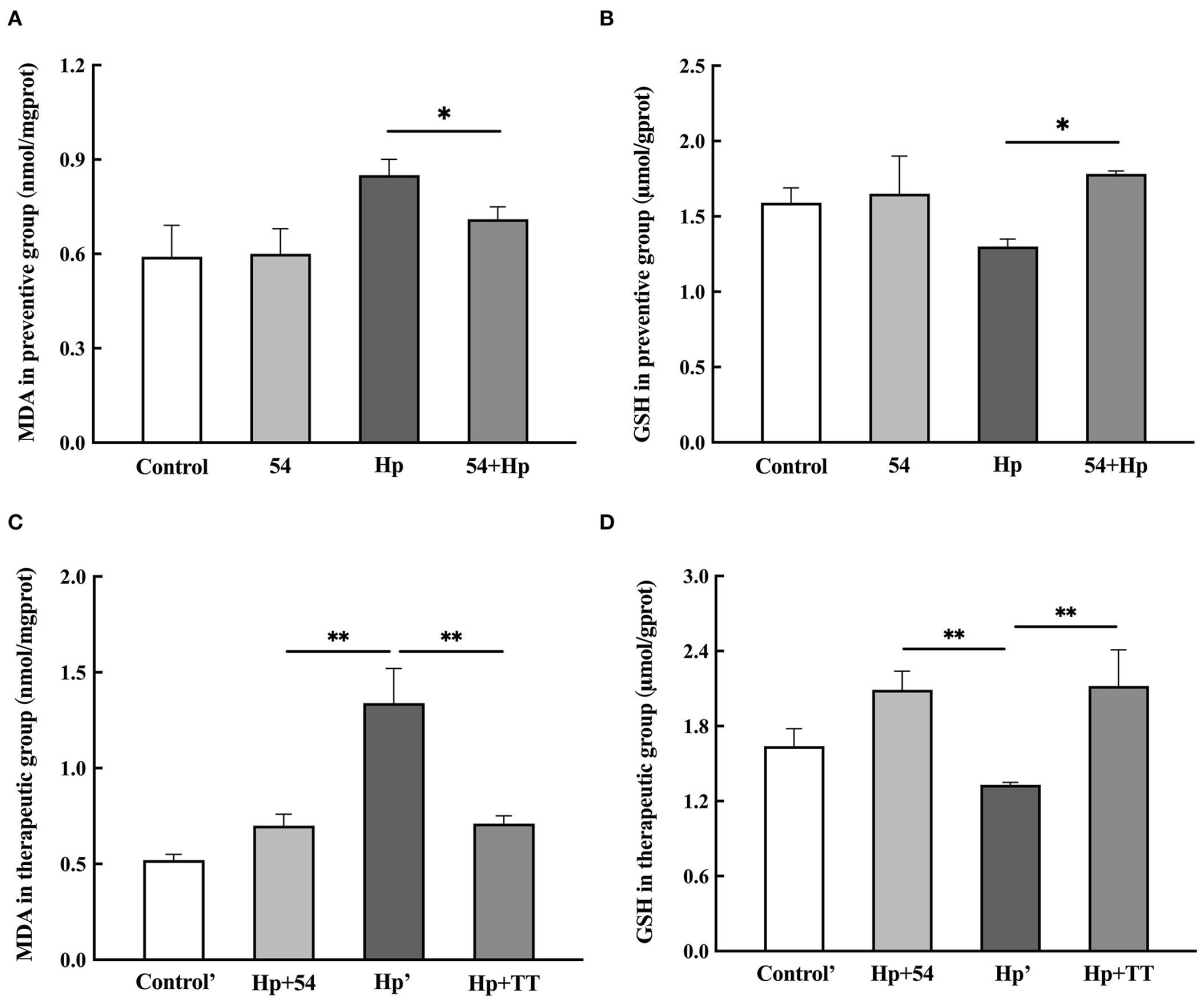
Levels of MDA and GSH during prevention and therapy. **(A)** MDA levels in preventive groups. **(B)** GSH levels in preventive groups. **(C)** MDA levels in therapeutic groups. **(D)** GSH levels in therapeutic groups. Data are shown as the mean ± SD (*n* = 6). Significant differences between two groups based on Student's *t*-test, *p < 0.05, **p < 0.01.

The MDA level in liver tissue of *H. pylori*-infected mice in the preventive group (0.85 ± 0.05 nmol/mgprot) was higher than that of the control group (*p* < 0.05) (Figure 4A). Pretreatment with *L. paracasei* ZFM54 significantly reduced MDA to 0.71 ± 0.04 nmol/mgprot (*p* < 0.05), thus protecting the mice mucosal layer from oxidative damage of ROS. Treatment with *L. paracasei* ZFM54 also significantly reduced the MDA level to 0.70 ± 0.06 nmol/mgprot (*p* < 0.01), when MDA contents were also significantly reduced in mice treated with triple therapy (TT) (0.71 ± 0.04 nmol/mgprot, *p* < 0.01) (Figure 4B).

GSH content in liver tissues of *H. pylori*-infected mice significantly decreased (1.30 ± 0.05 μmol/gprot) compared to control (1.59 ± 0.10 μmol/gprot, *p* < 0.05) (Figure 4C), while significant increased level of GSH was observed in mice pretreated with *L. paracasei* ZFM54 (1.78 ± 0.02 μmol/gprot, *p* < 0.05), treated with *L. paracasei* ZFM54 (2.09 ± 0.15 μmol/gprot, *p* < 0.01), and triple therapy (TT) (2.12 ± 0.29 μmol/gprot, *p* < 0.01) (Figure 4D).

### Histopathological changes of gastric tissues

**Figure 5:**
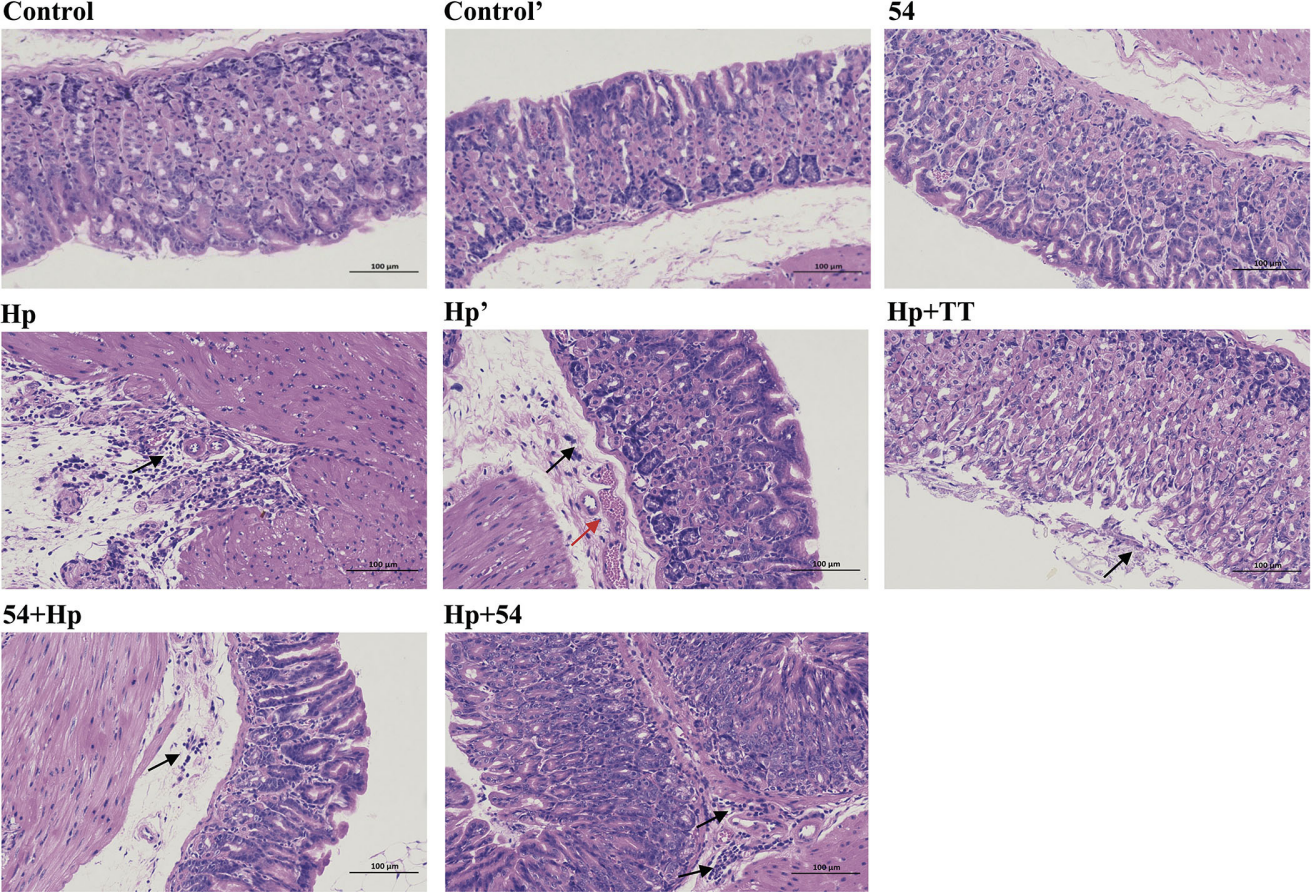
Hematoxylin–eosin staining of the gastric tissues. Control, normal mice without prevention and infection in the preventive group; Control', normal mice without infection and therapy in the therapeutic group; Hp, mice infected with *H. pylori* without prevention in the preventive group; Hp', mice infected with *H. pylori* without therapy in the therapeutic group; 54+Hp, pretreatment with *L. paracasei* ZFM54 before *H. pylori* challenge; Hp+54, treatment with *L. paracasei* ZFM54 after *H. pylori* challenge; Hp+TT, treatment with triple therapy after *H. pylori* challenge. Black arrow, infiltration of inflammatory cells in lamina propria; red arrow, vascular congestion in the submucosa.

Mice in the control and control' groups exhibited normal appearance of gastric mucosal morphologically, including the normal glands and structure of gastric epithelial cells, and little inflammatory infiltration in gastric epithelium (Figure 5). In contrast, the gastric mucosa of *H. pylori*-infected mice (Hp and Hp') showed irregular arrangement of mucosal glands, enlarged and hyperchromatic nuclei, and severe infiltration of inflammatory cells in the lamina propria. *L. paracasei* ZFM54 did not induce mucosal inflammation and other pathological changes in stomach. Inflammatory infiltration was significantly inhibited in mice pretreated with *L. paracasei* ZFM54 before *H. pylori* challenge (54+Hp). As expected, no obvious signs of tissue lesion and inflammation were also observed in the therapeutic groups of *L. paracasei* ZFM54 (Hp+54) and triple therapy (Hp+TT). These observations suggest that *L. paracasei* ZFM54 can inhibit or even reverse the injury process and protect gastric mucosa.

### mRNA expression level of inflammatory cytokines

**Figure 6:**
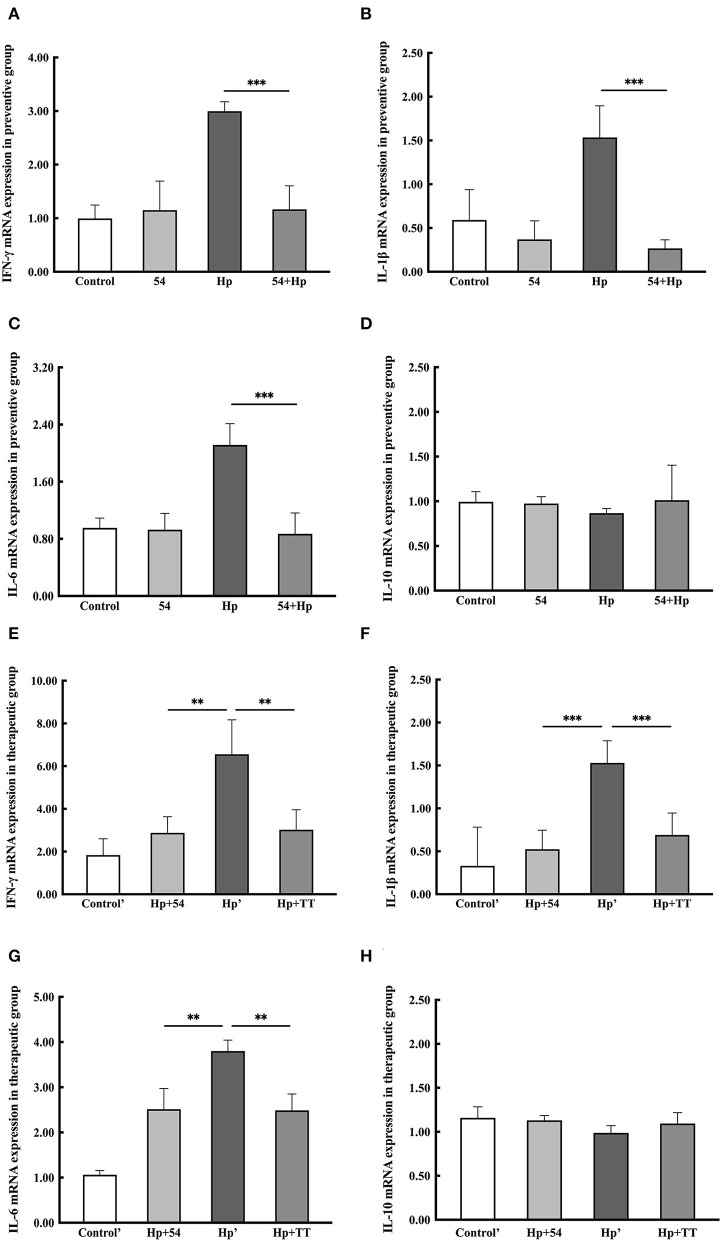
Expression levels of mRNA for pro-inflammatory or anti-inflammatory cytokines in preventive and therapeutic groups. **(A–D)** IFN-γ, IL-1β, IL-6, and IL-10 mRNA expression levels in the preventive group. **(E–H)** IFN-γ, IL-1β, IL-6, and IL-10 mRNA expression levels in the therapeutic group. Data are shown as the mean ± SD (*n* = 6). Significant differences between two groups based on Student's *t*-test, **p < 0.01, ***p < 0.001.

The levels of pro-inflammatory cytokines (IFN-γ, IL-1β, and IL-6) and anti-inflammatory cytokine (IL-10) were significantly altered after *H. pylori* infection, as displayed in Figure 4. In groups of Hp and Hp', the upregulation levels ranged between 3.00 ± 0.18 and 6.55 ± 1.86 log fold change of the INF-γ gene after *H. pylori* challenge, respectively (Figures 6A,E). IL-1β gene expression was significantly stimulated to 1.54 ± 0.36 and 1.53 ± 0.26 log folds, respectively (Figures 6B,F). Another pro-inflammatory cytokine, IL-6, was also increased to 2.11 ± 0.30 and 3.80 ± 0.24 log folds, respectively (Figures 6C,G). All pro-inflammatory cytokines genes were notably downregulated in *L. paracasei* ZFM54 prevention (54+Hp) and therapy (Hp+54) groups compared to probiotically unprotected mice (*p* < 0.01). No changes in the anti-inflammatory cytokine IL-10 were observed in either group (Figures 6D,H).

### Difference analysis of gastric microbiota

**Figure 7:**
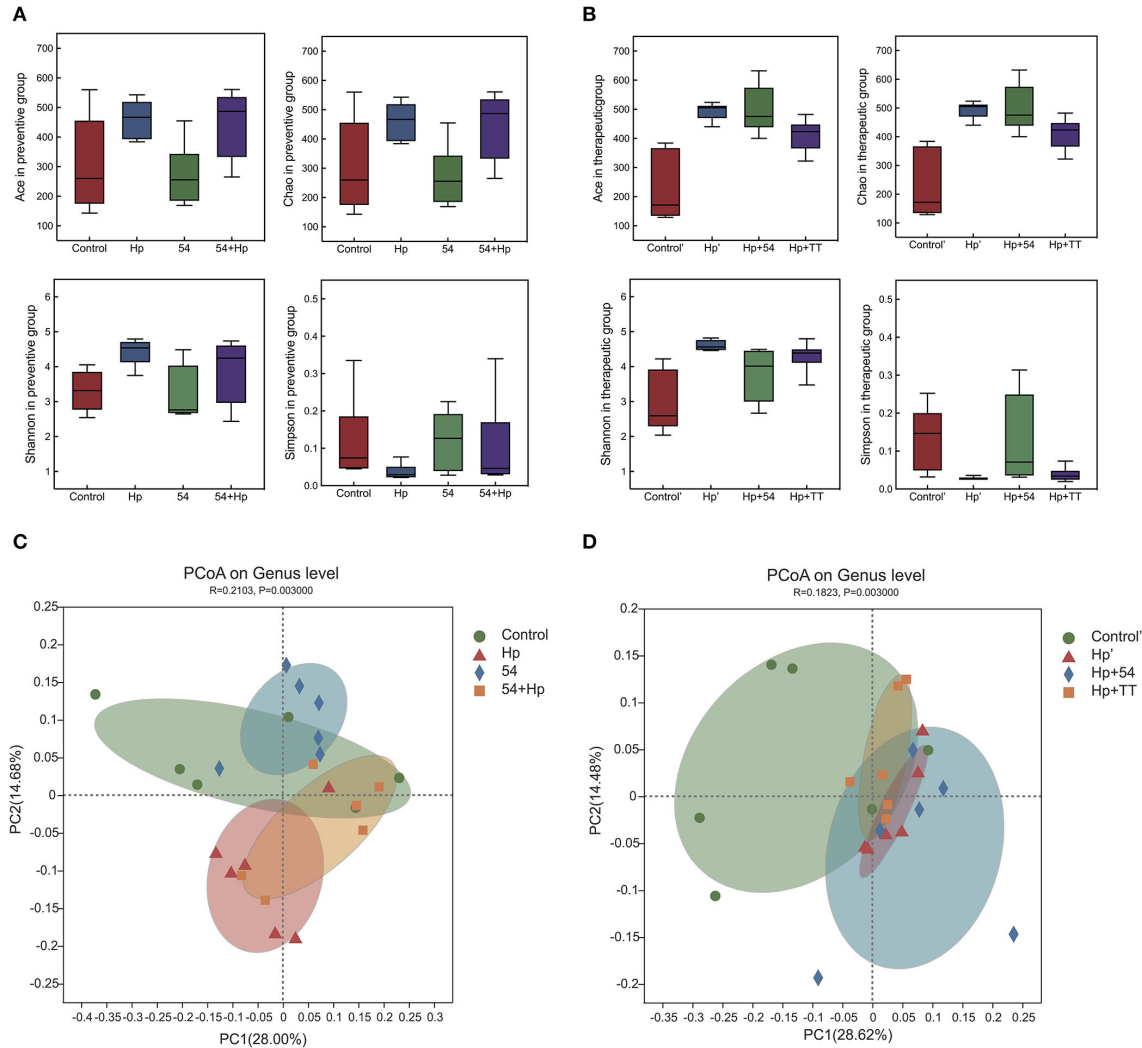
Bacterial alpha and beta diversity in the preventive and therapeutic groups. **(A)** Alpha diversity (Ace, Chao, Shannon, and Simpson) in the preventive group. **(B)** alpha diversity (Ace, Chao, Shannon, and Simpson) in the therapeutic group. **(C)** PCoA on genus level in the preventive group. **(D)** PCoA on the genus level in the therapeutic group.

To compare the gastric microbial composition in various groups of mice, we randomly selected the gastric contents for 16S high-throughput sequencing and taxonomic classification analysis. *H. pylori* infection increased the abundance and diversity of gastric microbiota by analysis of the Ace, Chao, Shannon, and Simpson indices of alpha diversity (Figures 7A,B). PCoA analysis showed apparent clustering of gastric microbiota in different groups based on genus-level unweighted-uniFrac distances (Figures 7C,D). Preventive and therapeutic administration of *L. paracasei* ZFM54 improved gastric microbiota structure to near normal levels.

**Figure 8:**
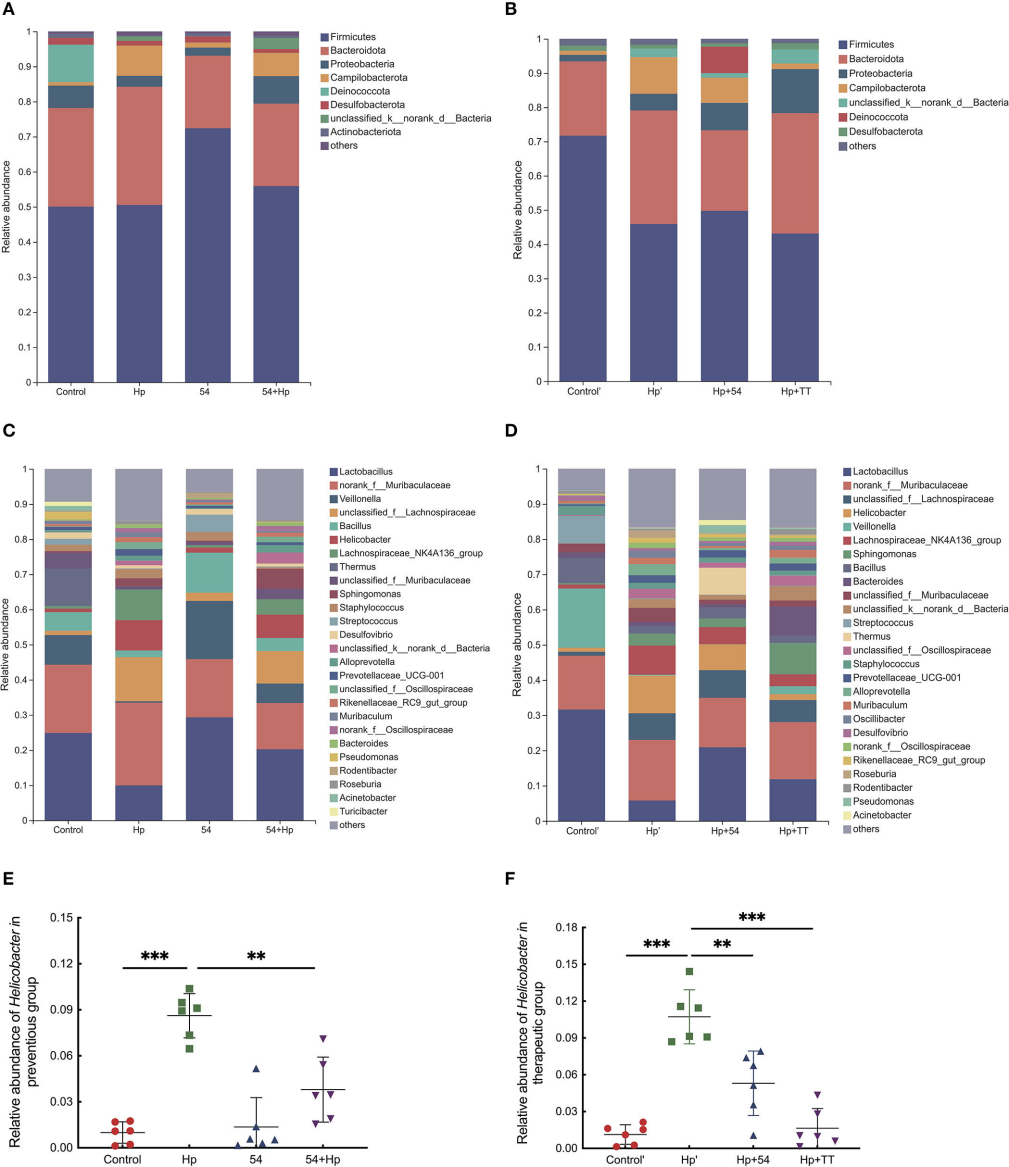
Bacterial community distribution among different groups at the phylum and genus levels. **(A)** Distribution of abundant phyla in the preventive group. **(B)** Distribution of abundant phyla in the preventive group. **(C)** Distribution of abundant genera in the preventive group. **(D)** distribution of abundant genera in the therapeutic group. **(E)** The relative abundance of *Helicobacter* in the preventive group. **(F)** The relative abundance of *Helicobacter* in the therapeutic group. Phyla with a mean abundance <1.0% and genera with a mean abundance <1.5% among all samples were consolidated. **p < 0.01, ***p < 0.001.

At the phylum level, it was found that the gavage of *L. paracasei* ZFM54 significantly increased the relative abundances of Firmicutes and Actinobacteriota and significantly decreased the abundance of Bacteroidota and Campilobacterota (Figures 8A,B). Instead, triple therapy treatment promoted the growth of Bacteroidota and Proteobacteria (Figure 8B). At the genus level, the relative abundances of bacterial groups such as *Lactobacillus, Veillonella*, and *Bacillus* in the *H. pylori* group decreased significantly, while *Helicobacter, Lachnospiraceae_NK4A136_group, Sphingomonas, Staphylococcus, Alloprevotella*, and *Prevotellaceae_UCG-001* increased significantly (Figures 8C,D). Compared with infected mice, *L. paracasei* ZFM54 prevention and treatment significantly decreased the relative abundance of *Helicobacter, Lachnospiraceae_NK4A136_group, Staphylococcus*, and *Prevotellaceae_UCG-001* and increased *Lactobacillus, Veillonella*, and *Thermus*. Although triple therapy treatment significantly inhibited the growth of gastric *Helicobacter*, it resulted in a significant increase in relative abundances of *Sphingomonas, Bacteroides, Streptococcus, Oscillibacter*, and *Desulfovibrio* (Figure 8D). The relative abundance of *Helicobacter* of 54, 54+Hp, Hp+54, and Hp+TT groups was lower than that of the *H. pylori* group (Hp and Hp', *p* < 0.01) (Figures 8E,F).

**Figure 9:**
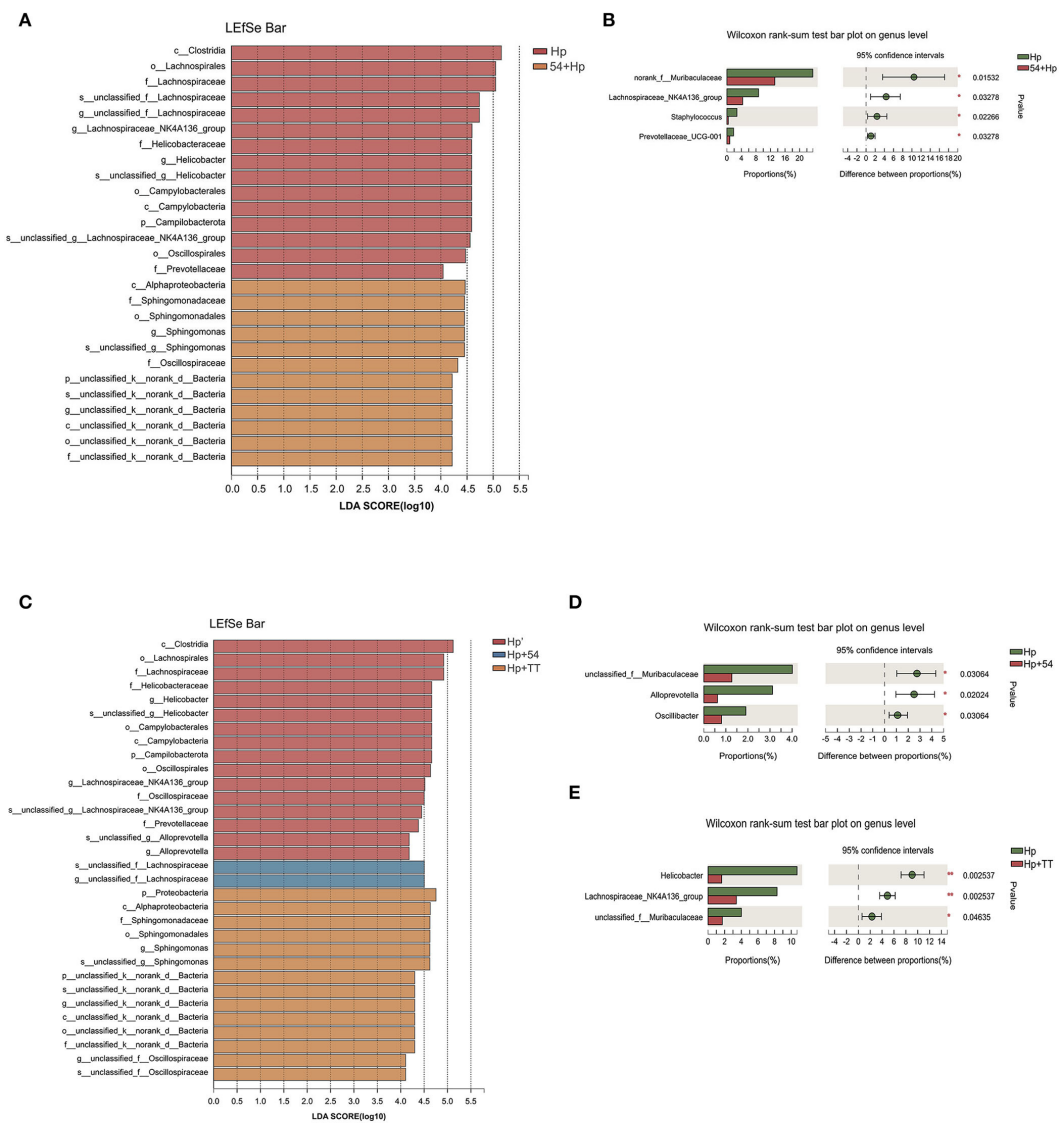
Difference in bacterial taxa among preventive and therapeutic groups. **(A)** Bacterial taxa significantly differentiated among the preventive group identified by the linear discriminant analysis (LDA). **(B)** Bacterial communities with significant relative abundance differences at the genus level between Hp and 54+Hp groups by the Wilcoxon rank-sum test (*n* = 6). **(C)** Bacterial taxa significantly differentiated among the therapeutic group by LDA. **(D)** Bacterial communities with significant relative abundance differences at the genus level between Hp and Hp+54 and Hp and Hp+TT groups by the Wilcoxon rank-sum test (*n* = 6). Only taxa meeting an LDA significance threshold of > 4 were shown, *p < 0.05, **p < 0.01.

Gastric microbiota in the normal, Hp infection, and intervention groups were compared using LEfSe. Abundant bacterial taxa observed in the Hp group were Lachnospiraceae, Helicobacteraceae, and Prevotellaceae, most of which belong to gram-negative human opportunistic pathogens (Figure 9A). Bacterial taxa were observed in abundance in pretreatment with *L. paracasei* ZFM54 including Sphingomonadaceae and Oscillospiraceae. According to the analysis of the Wilcoxon rank-sum test, significant reductions in the average relative abundance of *Muribaculum, Staphylococcus, Lachnospiraceae_NK4A136_group*, and *Prevotellaceae_UCG-001* were observed in the preventive group of *L. paracasei* ZFM54 on genus level compared to the Hp group (Figure 9B). In therapeutic groups (Figure 9C), 16 bacterial taxa were identified to be in abundance in the *H. pylori* group including Helicobacteraceae, Lachnospiraceae, Oscillospiraceae, and Prevotellaceae. Lachnospiraceae were observed in significant abundance in treatment with *L. paracasei* ZFM54 (Hp+54), while Sphingomonadaceae were abundant in triple therapy group (Hp+TT). In addition, significant reductions in average relative abundance of *Muribaculaceae, Alloprevotella*, and *Oscillibacter* were observed by *L. paracasei* ZFM54 treatment (Hp+54) (Figure 9D), while *Helicobacter, Lachnospiraceae_NK4A136_group*, and *Muribaculaceae* were significantly reduced by triple therapy treatment (Hp+TT) on the genus level using the Wilcoxon rank-sum test (Figure 9E).

## Discussion

Usually, host innate and adaptive immune responses are stimulated shortly after *H. pylori* colonization of gastric epithelial cells (27). Various clinical complications of *H. pylori* infection can be attributed to changes in bacterial genotypic and phenotypic characteristics. Infection with *vacA*- and *cagA*-positive *H. pylori* leads to severe gastric inflammation and a high risk for cancer development (28). *H. pylori* ZJC03 in this study was previously isolated from a patient with gastritis, which has virulence genes of *vacA* and *cagA* and is highly resistant to metronidazole. *Lactobacillus* strains are among the most prominent members of probiotics, mostly normal residents of different regions of human body. Our previous study examined the prebiotic properties of *L. paracasei* ZFM54 by genome sequence analysis and related prebiotic properties, including antagonism against *H. pylori* ZJC03, antibiotic susceptibility, and no adverse effects during acute oral toxicity in mice (29–31), suggesting that *L. paracasei* ZFM54 can be a potential candidate for use in the health or diet field.

For an effective strain to be called a probiotic, it must be able to survive in a hostile gastric environment (32). To reach host epithelial tissues of stomach, the acidic pH (1–2) in stomach is the foremost challenge to overcome. The ingested food buffers the acidic environment to pH 3, so it is considered as the optimum pH for the survival of a potential probiotic strain (33). *L. paracasei* ZFM 54 exhibited good tolerance as it showed considerable growth at low pH (2.0). Sornsenee et al. found that the survival rates of eight *L. paracasei* strains isolated from fermented palm sap in Thailand were 40–60% at pH 2 for 4 h (34). *L. paracasei* L1 was highly resistant to low pH (viability of 98.73% at pH 2 for 3 h) and was fed as probiotics to improve the intestinal microflora of chicken (35). The auto-aggregation ability of a probiotic strain is another important factor correlated with the bacterium's ability to form biofilm, which is an important property of probiotic to prevent pathogenic microorganisms from invading the host (1). The auto-aggregation rate of *L. paracasei* ZFM 54 rose to 69.00 ± 2.73% after 4 h. The 2- and 24-h auto-aggregation values of *L. paracasei* L1 were 20.4 ± 2.3% and 47.2 ± 0.8%, respectively (35). Co-aggregation is considered a key strategy for *Lactobacillus* strains to prevent pathogenic bacteria from adhering to biological surfaces (36). Barache et al. analyzed that *L. paracasei* FB1 showed stronger co-aggregation with *S. aureus* (28.34% ± 2.56%) after 2 h of incubation, followed by *E. coli* (23.39 ± 2.12%) and *L. monocytogenes* (20.10 ± 2.04%) (37). *L. paracasei* ZFM 54 displayed a co-aggregation rate of over 30% and exhibited their capability to agglutinate *H. pylori in vitro*.

Inhibition of urease enzyme is an important ability of *Lactobacillus* to decrease *H. pylori* density in the host and inhibit dysbiosis. *H. pylori* uses urease enzyme to survive in the acidic environment of the stomach by utilizing urease enzyme activity to convert urea into ammonia, thus raising the pH and creating a favorable environment for its growth and colonization (38). Infection with *H. pylori* stimulates phagocytic cells at inflammation sites, producing ROS to combat *H. pylori* (17). The increased level of ROS is detrimental for host tissues, causes lipid peroxidation, and produces MDA. MDA is a biomarker of *H. pylori* infection (17). GSH, a thiol-based anti-oxidant, is reduced in *H. pylori* infection, which is naturally present in all cells and contributes in defending against oxidative injury. As observed in this study, *L. paracasei* ZFM 54 significantly reduced *H. pylori*-mediated MDA level in mice liver tissues, while significantly increasing the GSH level in preventive and therapeutic studies. *L. paracasei* ZFM 54 appears to have potential anti-oxidation ability *in vivo* as well, validating the anti-oxidation potential shown in *in vitro* experiment.

*H. pylori* infection stimulates adaptive immune response mainly through the activation of T-helper cells. Th1 cells further produce cytokines IL-2, IL-22, and IFN-γ with subsequent stimulation of neutrophil infiltration and macrophage activation (39). *H. pylori* modulate pro-inflammatory and regulatory immune responses, which stimulate the activation of IL-1β, a strong pro-inflammatory cytokine which promotes gastric tumor cancer through genetic polymorphism (40). Consistent results in this study were observed through *H. pylori-*mediated enhanced level of pro-inflammatory cytokines (IFN-γ, IL-1β, and IL-6) and severe infiltration of inflammatory cells in gastric mucosa. Prevention and therapy with *L. paracasei* ZFM 54 significantly downregulated the pro-inflammatory cytokines in gastric mucosa. Previously, similar results were obtained in pretreatment with *L. plantarum* ZDY 2013 against *H. pylori* in mice (41). *L. plantarum* ZDY 2013 showed a significant increase in IL-10 against *H. pylori* in AGS cell lines (42). No increase in the level of anti-inflammatory cytokine IL-10 was observed in this research, suggesting the different mechanisms to be involved in reducing inflammation. Other mechanisms responsible for the protective effect may be contributed by competitive exclusion of *H. pylori* or inhibition by secreting antimicrobial substances or blocking urease activity, which further hinder *H. pylori* colonization.

The microbiota in normal healthy stomach has been reported to be mainly composed of Firmicutes, Bacteroidetes, Actinobacteria, and Proteobacteria, and the most abundant genera have been reported to include *Streptococcus, Prevotella, Fusobacteria, Veillonella*, and *Rothia* (43, 44). Previous reports provided evidence of reduced bacterial diversity and an increase in the bacterial taxa belonging to the phyla Campilobacterota and Proteobacteria in subjects with *H. pylori-*positive infection (43). Preventive and therapeutic experiments with *L. paracasei* ZFM 54 significantly suppressed the relative abundance of *H. pylori*, while increasing the abundance of bacterial taxa belonging to the phyla Firmicutes and Actinobacteriota. Interestingly, treatment with triple therapy reduced high levels of inflammation cytokines and MDA, but the LEfSe analysis showed increased abundance of *Rodentibacter* and *Escherichia-Shigella Mucispirillum*, members of which are opportunistic human pathogens in the stomach. It is also suggestive of disruption of gastric microbiota due to antibiotics. Suppression of acid by PPI triggers dysbiosis in gastric microbiota composition (45). An increased abundance of non-*H. pylori* bacteria such as *Escherichia-Shigella, Nitrospirae*, and *Burkholderia* were found in patients with gastric cancer (46).

## Conclusion

The strain *L. paracasei* ZFM 54 demonstrated remarkable probiotic characteristics owing to its ability to survive in the simulated gastric acid, auto-aggregation, and hydrophobic activities. The potential inhibition against *H. pylori* and urease enzyme, anti-oxidizing, and co-aggregation activities further validate the efficacy of the strain to be effective against *H. pylori*. During preventive and therapeutic experiments in the *H. pylori*-induced gastritis mouse model, *L. paracasei* ZFM 54 can effectively reduce inflammation, MDA level, and inhibition of microbiota dysbiosis. The use of *L. paracasei* ZFM 54 against *H. pylori* challenge would be more advantageous over antibiotics, as it more favorably improves gastric microbiota caused by *H. pylori* infection. The potential to prevent and treat *H. pylori* inflammation is highly indicative of its use not only as a prophylaxis but also as a therapeutic agent to eradicate *H. pylori*.

## Data availability statement

The original contributions presented in the study are included in the article, further inquiries can be directed to the corresponding author.

## Ethics statement

The animal study was reviewed and approved by mice *in vivo* Experiments was conducted under approval of by Shanghai Public Health Clinical Center Laboratory Animal Welfare and Ethics Committee with protocol No. 2019-A003-01.

## Author contributions

PL and QG: conceptualization and supervision. QZ, NQ, and BX: methodology, investigation, and data curation. QZ and NQ: writing—original draft preparation. QZ and ZX: writing—review and editing. QG: funding acquisition. All authors have read and agreed to the published version of the manuscript.

## Funding

The work was supported by the Chinese Academy of Engineering Academy-Locality Cooperation Project (No. 2019-ZJ-JS-02) and the Natural Science Foundation of Zhejiang Province of China (No. LZ21C200001).

## Conflict of interest

The authors declare that the research was conducted in the absence of any commercial or financial relationships that could be construed as a potential conflict of interest.

## Publisher's note

All claims expressed in this article are solely those of the authors and do not necessarily represent those of their affiliated organizations, or those of the publisher, the editors and the reviewers. Any product that may be evaluated in this article, or claim that may be made by its manufacturer, is not guaranteed or endorsed by the publisher.
